# Immunohistological detection of *Chlamydia pneumoniae *in the Alzheimer's disease brain

**DOI:** 10.1186/1471-2202-11-121

**Published:** 2010-09-23

**Authors:** Christine J Hammond, Loretta R Hallock, Raymond J Howanski, Denah M Appelt, C Scott Little, Brian J Balin

**Affiliations:** 1 Pathology/Microbiology/Immunology and Forensic Medicine Department, Philadelphia College of Osteopathic Medicine, 4170 City Ave, Philadelphia, Pennsylvania, USA; 2 Neuroscience/Physiology/Pharmacology Department, Philadelphia College of Osteopathic Medicine, 4170 City Ave, Philadelphia, Pennsylvania, USA; 3 Center for Chronic Disorders of Aging, Philadelphia College of Osteopathic Medicine, 4170 City Ave, Philadelphia, Pennsylvania, USA

## Abstract

**Background:**

Sporadic late-onset Alzheimer's disease (AD) appears to evolve from an interplay between genetic and environmental factors. One environmental factor that continues to be of great interest is that of *Chlamydia pneumoniae *infection and its association with late-onset disease. Detection of this organism in clinical and autopsy samples has proved challenging using a variety of molecular and histological techniques. Our current investigation utilized immunohistochemistry with a battery of commercially available anti-*C. pneumoniae *antibodies to determine whether *C. pneumoniae *was present in areas typically associated with AD neuropathology from 5 AD and 5 non-AD control brains.

**Results:**

Immunoreactivity for *C. pneumoniae *antigens was observed both intracellularly in neurons, neuroglia, endothelial cells, and peri-endothelial cells, and extracellularly in the frontal and temporal cortices of the AD brain with multiple *C. pneumoniae*-specific antibodies. This immunoreactivity was seen in regions of amyloid deposition as revealed by immunolabeling with two different anti-beta amyloid antibodies. Thioflavin S staining, overlaid with *C. pneumoniae *immunolabeling, demonstrated no direct co-localization of the organism and amyloid plaques. Further, the specificity of *C. pneumoniae *labeling of AD brain sections was demonstrated using *C. pneumoniae *antibodies pre-absorbed against amyloid β 1-40 and 1-42 peptides.

**Conclusions:**

Anti-*C. pneumoniae *antibodies, obtained commercially, identified both typical intracellular and atypical extracellular *C. pneumoniae *antigens in frontal and temporal cortices of the AD brain. *C. pneumoniae*, amyloid deposits, and neurofibrillary tangles were present in the same regions of the brain in apposition to one another. Although additional studies are required to conclusively characterize the nature of Chlamydial immunoreactivity in the AD brain, these results further implicate *C. pneumoniae *infection with the pathogenesis of Alzheimer's disease.

## Background

Alzheimer's disease (AD) is a progressive neurological disease that affects millions of older individuals. Distinctive pathological hallmarks associated with the disease include tau accumulations forming neuropil threads (NTs) and neurofibrillary tangles (NFTs), and deposits of extracellular amyloid comprising neuritic senile plaques (NSPs). In general, there are two distinct forms of Alzheimer's disease, familial AD and sporadic late-onset AD. The early onset form of the disease, known commonly as familial AD, is caused by dysregulation of many processes due to genetic mutations that lead to the aforementioned pathology. For example, mutations in presenilins 1 and 2 genes and the gene responsible for amyloid-β protein precursor (AβPP) result in an increased accumulation of beta-amyloid (Aβ) in the brain. In late-onset sporadic AD, similar pathological accumulations occur, although without the gene mutations seen in familial AD (for review see Duyckaerts 2009) [1].

Most investigations have focused on the extracellular, deposited forms of amyloid in the AD brain. The extracellular accumulations of amyloid in the brain are composed principally of amyloid β 1-40 and 1-42 and form neuritic senile plaques (NSP) [1,2]. However, intracellular accumulations of amyloid, which may occur prior to extracellular deposition, also have been demonstrated [3-6]. Further, Aβ-derived diffusible ligands (ADDLs), soluble forms of amyloid, have been postulated to be a toxic form of amyloid at the synapses and are not found in typical neuropathological or histopathologic accumulations of amyloid [7]. Since late-onset AD lacks the same mutations seen in familial AD, determination of the cause of amyloid pathology in late-onset AD remains poorly understood.

The interplay between normal processes and environmental factors, both independently and in concert with other genetic factors, may lead to late-onset AD. In particular, infections as environmental factors may have an impact on the delicate amyloid and tau balance in the brain and lead to the pathology seen in AD. A number of infectious agents have been associated with late-onset AD [8-12]. Our focus has been on the obligate, intracellular bacterium *Chlamydia pneumoniae*, which has been demonstrated to be highly prevalent in the AD brain [8,12], as well as associated with other systemic and neurological diseases [13,14]including atherosclerosis [15,16], stroke [17], encephalitis [18], and multiple sclerosis [19].

Various cell types found in the brain have been shown to be susceptible to infection by *C. pneumoniae *including endothelia, astroglia, microglia, and neurons [8,12,20-23]. Once inside the cell, *C. pneumoniae *reside in an intracellular inclusion that resists lysosomal fusion and immune recognition. *C. pneumoniae *developmentally cycle from the infectious elementary body (EBs) to the metabolically active reticulate body (RBs), which divide by binary fission. This obligate intracellular pathogen both interacts with and manipulates the host by gathering energy and nutrients that are required for replication, such as sphingomyelin and cholesterol [24-26]. Chlamydiae also inhibit apoptosis [20,27-29] and release factors such as chlamydial lipopolysaccharide (LPS) and glycolipid protein into both the cell itself and into the surrounding milieu [30]. Further propagation and spread of the organism may follow one or more pathways. The infectious progeny, EBs, may be released upon eventual cell death or by extrusion from the cell in a membrane bound package into the surrounding environment [31]. Thus, *C. pneumoniae *and/or antigens derived from the organism may be localized both intracellularly and extracellularly at the site of infection.

Due to the chronic nature of AD and the complexity of *C. pneumoniae *infections, establishing an association with disease pathogenesis has proved difficult. Validating this association relies on a variety of detection methods for the organism. Our current study focuses on the use of immunohistochemistry (IHC) with a battery of commercially available anti-chlamydia antibodies on frontal and temporal cortical sections of human AD brains. Our data suggest this methodology provides a valuable insight into the interrelationship between infection and AD pathology.

## Results

### *C. pneumoniae *immunolabeling in AD tissue

**Figure 1:**
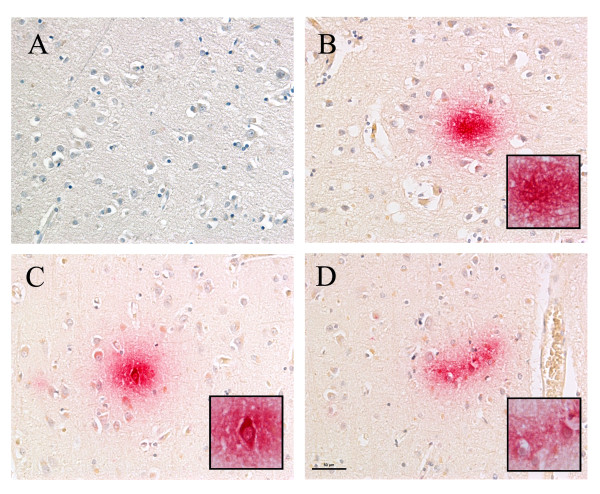
***Chlamydia pneumoniae *immunoreactivity in AD brain tissue**. Alzheimer's disease brain cerebral cortex immunolabeled with a mouse anti-***C. pneumoniae ***monoclonal antibody (Table 1, #5) demonstrates both intracellular and extracellular immunolabeling as indicated by the magenta color. Panel A illustrates the temporal cortex labeled with no primary antibody and both anti- mouse HRP and anti-mouse AP conjugated secondary antibodies reacted with both DAB and AP red. Panel B illustrates an extracellular labeling pattern and panels C and D illustrate cellular labeling in the frontal cortex. Areas delineated by the boxes are higher magnification images of the immunoreactivity in panels B-D. Size bar = 50 μm.

**Figure 2:**
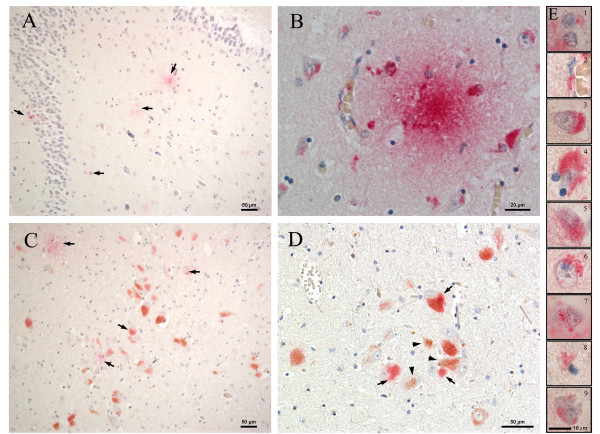
***Chlamydia pneumoniae *immunoreactivity in hippocampus and entorhinal Cortex**. Detection of ***C. pneumoniae ***antigen in the Alzheimer's disease hippocampus in panels A and B. Representative immunolabeling with mouse anti-***C. pneumoniae ***monoclonal antibody (Table 1, #5) shows immunopositivity in the dentate gyrus of the hippocampus. Immunolabeling is indicated by the magenta color and is denoted by the arrows in panel A. Extracellular and intracellular ***C. pneumoniae ***immunolabeling in apparent large neurons is found within the entorhinal cortex, panels C and D. Prominent labeling (magenta) denoted by the arrows in panels C and D, is easily distinguished from intraneuronal lipofuscin (golden brown) denoted by the arrowheads (panel D). High magnification images of chlamydial labeling within different cell types in the hippocampal region are illustrated in panel E (1, 8: neuroglia; 2: peri-vascular; 3, 4, 5, 6, 7, 9: neuronal). Note the apparent tangle within the neuron in E9.Size bars: A, C, and D = 50 μm; B = 20 μm; E = 10 μm.

All available sections from the frontal and temporal cortices of AD and control brains were immunolabeled with anti-*C. pneumoniae *antibodies listed in table 1. All AD brains were found to immunolabel with all of the anti-*C. pneumoniae *antibodies, although the type of labeling profile (eg, intracellular versus extracellular) and extent of label differed. Representative labeling profiles were highlighted in Figure 1. An AD brain immunolabeled with no primary antibody and both an anti-mouse horseradish peroxidase (HRP) conjugated secondary and an anti-mouse alkaline phosphatase (AP) conjugated secondary reacted with both 3, 3′-Diaminobenzidine (DAB) and AP red illustrate the absence of non specific immunolabeling in the temporal cortex. The AD frontal cortex labelled with a mouse anti-*C. pneumoniae *monoclonal antibody (Table 1, #5) demonstrated both neuropil (Figure 1B) and intracellular (Figure 1C, D) immunolabeling. *C. pneumoniae *was present in the hippocampus and entorhinal cortex of Alzheimer's disease brain (Figure 2). Representative immunolabeling with an anti-*C. pneumoniae *monoclonal antibody (Table 1, #5) was observed in the dentate gyrus of the hippocampus indicated by the magenta color (Figure 2A, B). *C. pneumoniae *immunolabeling also was observed in apparent large neurons within the entorhinal cortex (Figure 2C, D) that also contained intraneuronal lipofuscin accumulations (golden brown, arrowheads). Although the chlamydia immunoreactivity was detected using all anti-chlamydia antibodies in all AD brains, the extent of the label varied with the antibody used and AD case examined. Chlamydia immunolabeling was detected in the frontal and temporal cortices and appeared distributed across all 6 cortical layers. Although the immunolabeling may appear robust in some areas, in general, the intracellular immunoreactivity was observed in less than 1% of brain cells in these regions. The cell types in the grey matter within the hippocampal formation (Figure 2E) that were labeled intracellularly include neuroglia, large and small neurons, pyramidal neurons, although less commonly, and peri-vascular cells. Similar cell types showed immunoreactivity across all frontal and temporal cortical layers. This intracellular immunoreactivity was occasionally seen in cells with granulovacuolar degeneration and tangles in these regions. Additionally, the more atypical extracellular chlamydia immunoreactivity was visualized across all cortical layers with approximately 1-2% of the area in a chlamydia-positive field demonstrating immunoreactivity. This varies widely with different AD cases. Further, the immunoreactivity was seen in the white matter and even the cerebellum although study of this immunolabeling was outside the scope of this report. Minimal immunoreactivity was seen in the 2 of 5 non AD cases in the frontal and temporal regions, not confined to any specific layer; and there was some hippocampal involvement.

**Table 1:** Commercially available Chlamydia antibodies

1	BioDesign, Meridian Life Sciences	B65256R	Polyclonal	1:100
2	BioDesign, Meridian Life Sciences	C65165M	Monoclonal	1:100

3	BioDesign, Meridian Life Sciences	C65691M	Monoclonal	1:100

4	Fitzgerald	10-C27	Monoclonal	1:100

5	GenWay Biotech	20-902-170121	Monoclonal	1:100

6	GenWay Biotech	20-272-190984	Monoclonal	1:100

### Amyloid and *C. pneumoniae *immunolabeling in AD tissue

**Figure 3:**
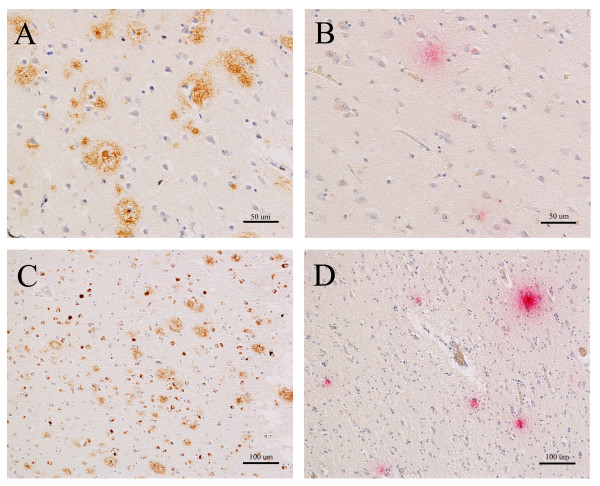
**Amyloid and *Chlamydia pneumoniae *immunolabeling in AD tissue**. Representative amyloid and ***C. pneumoniae ***immunolabeling detected in frontal (A, B) and temporal (C, D) sections of an AD brain. For amyloid labeling (brown color), two different anti-amyloid antibodies were used, (Sigma, panel A) and (Signet, panel C). Representative amyloid immunoreactivity, both extracellular plaques, including dense core plaques (A), and intracellular labeling (C) are revealed at a low magnification in order to appreciate the level of amyloid pathology. Likewise, ***C. pneumoniae ***immunoreactivity (magenta color) is evident at low magnification in both brain regions using two different monoclonal antibodies (Table 1, #5 panel B), and (Table 1, #3 panel D). Size bar A, B = 50 μm; C, D = 100 μm.

Amyloid and *C. pneumoniae *immunoreactivity were detected in sections of the AD frontal (Figure 3A, B) and temporal cortices (Figure 3C, D). Amyloid immunoreactivity was present in dense core mature plaques (brown color) using a rabbit anti-amyloid 1-42 antibody (Sigma) (Figure 3A), and a mouse monoclonal anti-amyloid antibody (4G8, Signet) which also demonstrated intraneuronal amyloid labeling in the temporal region of the AD brain (Figure 3C). Both the frontal and temporal cortices demonstrated *C. pneumoniae *intra- and extracellular immunoreactivity with multiple monoclonal antibodies (Figure 3B, D; Table 1, #5, #3, respectively). Both amyloid and chlamydial immunoreactivity were visible at low magnification. Interestingly, although *C. pneumoniae *was found in the frontal cortex in the AD cases, we observed more consistent evidence of *C. pneumoniae *in the temporal cortex. Further, Chlamydial immunoreactivity occurred in apposition with amyloid pathology in chlamydia positive areas. There was approximately 5-10% of the area immunolabeled with amyloid compared to 1-5% of the same area immunolabeled for intracellular and extracellular chlamydia. There appeared to be no consistent pattern to the area or region in which the immunolabeling occured. Furthermore, in the temporal cortex for 2 of 5 non-AD cases, *C. pneumoniae *labeling was observed, although less prominently as compared to the AD brains. These non AD cases also demonstrated diffuse amyloid immunopositivity (data not shown).

### Association of *C. pneumoniae *labeling and Thioflavin S staining

**Figure 4:**
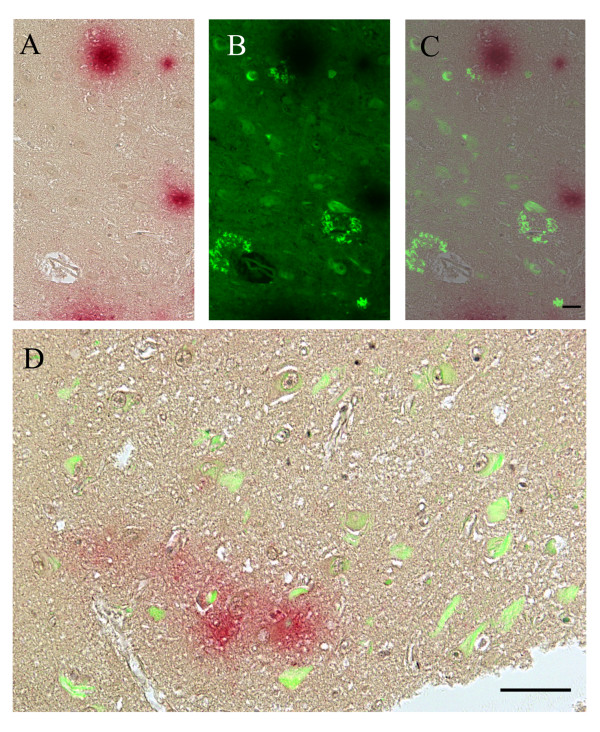
**Dual *Chlamydia pneumoniae *labeling and Thioflavin S staining of the temporal cortex from an AD brain **.Alzheimer's disease temporal cortex is immunolabeled with a monoclonal anti-***C. pneumoniae ***antibody (Table 1, #5) followed by staining with Thioflavin S on the same tissue specimen. Panel A shows intracellular and extracellular ***C. pneumoniae ***immunoreactivity (magenta color). Using a FITC filter, panel B shows both Thioflavin S positive intracellular labeling, presumably NFTs, and extracellular amyloid plaques (yellow fluorescence). Panel C shows the light (panel A) (***C. pneumoniae***) image overlaid on the fluorescent (panel B) (Thioflavin S) image. Panel D shows merged light and fluorescent images of another region of this temporal cortex illustrating merged chlamydia chromogen immunoreactivity and Thioflavin S amyloid/tau fluorescence. Size bars = 50 μm.

To further illustrate the relationship between amyloid and chlamydia, temporal AD sections were labeled with an anti-*C. pneumoniae *antibody (Table 1, #5) (red) and then stained for Thioflavin S (yellow-green fluorescence using the FITC filter ) on the same section. For this dual procedure, optimal results were obtained when immunohistochemistry (IHC) was performed prior to incubating with Thioflavin S. Both light and fluorescent images of the areas of interest were captured and then overlaid or merged. This approach revealed the close proximity of chlamydia with NFTs and NSPs, which are hallmarks of Alzheimer's disease pathology (Figure 4).

### Pre-absorption of anti-*C. pneumoniae *antibodies with Amyloid β 1-40 and 1-42 peptides

**Figure 5:**
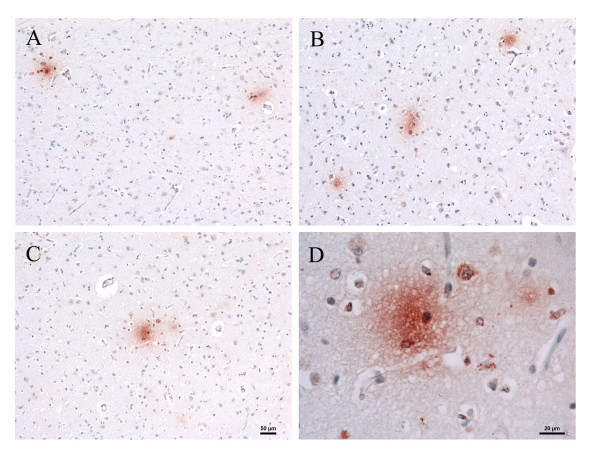
**Immunohistochemistry using an amyloid-pre-absorbed anti-*Chlamydia pneumoniae *antibody**. Panels A-D illustrate anti-***C. pneumoniae ***immunoreactivity (red) in frontal AD tissue. The antibody for detection of ***C. pneumoniae ***is a mouse monoclonal ***C. pneumoniae ***antibody (Table 1, #3). Panel A illustrates the extent of anti-***C. pneumoniae ***immunoreactivity with the non-pre-absorbed antibody. Panel B shows the extent of immunolabeling on the tissue when labeled with the 1-40 pre-absorbed antibody (pre-absorbed against >10 molar excess Sigma amyloid Aβ1-40 peptide). Panels C and D (higher power of panel C) show the extent of immunolabeling on the tissue when labeled with the 1-42 pre-absorbed antibody (pre-absorbed against >10 molar excess Sigma amyloid Aβ1-42 peptide). Size bar for panels A-C = 50 μm and panel D = 20 μm.

Upon detection of atypical extracellular chlamydia immunoreactivity, we questioned whether anti-chlamydia antibodies could label extracellular deposits of amyloid. To address this issue, we labeled several AD brain serial sections with separate anti-*C. pneumoniae *antibodies that had been pre-absorbed with amyloid peptides. The immunoreactivity for *C. pneumoniae *was not depleted after labeling with these pre-absorbed antibodies (Figure 5A-D). Figure 5A demonstrated the representative extracellular and intracellular immunoreactivity of a non- pre-absorbed monoclonal anti-*C. pneumoniae *antibody (Table 1, #3) on frontal AD tissue. Similar extracellular and intracellular immunolabeling of *C. pneumoniae *antigen was obtained with the antibody pre-absorbed against >10 molar excess Sigma amyloid Aβ1-40 peptide (Figure 5B) and with the antibody pre-absorbed against >10 molar excess Sigma amyloid Aβ1-42 peptide (Figure 5C, D). Comparable results were obtained with two other anti-*C. pneumoniae *antibodies pre-absorbed in the same manner (Table 1, #1, #2) (data not shown).

## Discussion

Intracellular and extracellular *C. pneumoniae *immunoreactivity was observed in the entorhinal cortex, the hippocampal formation, and the frontal cortex, regions of the brain that typically demonstrate AD pathology. Clear discrimination between chlamydia immunoreactivity and age-related lipofuscin accumulation within neurons was demonstrated. Serial sections of brain tissue displayed both amyloid pathology and the presence of *C. pneumoniae *immunoreactivity. Thioflavin S staining for fibrillar amyloid and specific antibody labeling for *C. pneumoniae *revealed deposition of both when performed on the same section. As some *C. pneumoniae *labeling was extracellular, a more atypical pattern, pre-absorption studies with Amyloid β 1-40 and 1-42 were performed. These studies revealed that *C. pneumoniae *antibodies were not cross-reacting with Aβ. Collectively, these data demonstrate that evidence of *C. pneumoniae *infection is present in brain tissues in areas of amyloid pathology, thereby suggesting that an interrelationship exists between these entities in the pathogenesis of sporadic late-onset AD.

Immunolabeling for Chlamydia may be overlooked in brain tissues, as it is different from what is observed in cellular infections *in vitro*. In all AD samples analyzed in this study, both typical intracellular perinuclear chlamydia immunoreactivity and atypical extracellular labeling were observed. Intracellular labeling demonstrated punctate elementary bodies and membrane bound inclusions similar to that of *in vitro *studies (see Figure 2E). This specific labeling was differentiated from lipofuscin by using red chromogens, either alkaline phosphatase (AP)red or AP magenta, as the substrate to denote *C. pneumoniae *immunoreactivity. Horseradish peroxidase labeling with 3, 3′-Diaminobenzidine (DAB), a brown chromogen, was not used as this labeling may be confused with the golden brown lipofuscin found in neurons of aged brains.

Two distinct extracellular patterns of chlamydia immunoreactivity were observed: one, a punctate pattern signifying the elementary body form of the bacteria, which can be extruded from infected cells into the surrounding milieu [31], and two, an amorphous foci pattern most likely indicating secreted chlamydial factors such as lipopolysaccharide [30,31]. These patterns in the cerebrum will require further study although similar profiles of Chlamydia labeling *in situ *have been demonstrated in a different organ [32]. Furthermore, our data demonstrated that *C. pneumoniae *extracellular immunoreactivity was not reflective of cross-reactivity with extracellular Amyloid β 1-40 or 1-42. However, *C. pneumoniae *extracellular organism and related antigens may interact with extracellular proteins and lipids in the brain. Although not always in direct overlap with amyloid plaque deposits, chlamydial antigens may interact with soluble oligomeric forms of amyloid, such as ADDLs, that are less likely to be found in mature plaques due to their soluble nature [7]. These intriguing findings and their implications require further understanding of the possible relationship between amyloid and chlamydia in the same cortical regions of the brain. This relationship will vary with each individual AD patient. Each AD patient has different levels of pathology and may have corresponding variability in extent and distribution of *C. pneumoniae *infection in the cerebrum. Following further studies into this variability, the relationship between pathology and infection can be more thoroughly evaluated.

Although *C. pneumoniae *is principally a respiratory pathogen, infection of the brain has been shown following intranasal and lung infection [33,34]. In this regard, monocytes infected with *C. pneumoniae *in the lungs may spread the infection via the peripheral circulation to the brain through the blood brain barrier or circumventricular organs [22,23]. Alternatively, a more direct and insidious route of infection may follow the olfactory pathways. As such, the infection becomes established in the olfactory nasal neuroepithelia, progresses to the olfactory bulbs, and eventually infects brain structures such as the entorhinal cortex and hippocampus. The olfactory structures, the entorhinal cortex, and the hippocampal formation are the most vulnerable and the earliest regions affected in the onset of AD [35,36]. Our current study highlights *C. pneumoniae *detection in the frontal and temporal cortices, including the entorhinal cortex and the hippocampal formation. Thus, infection of these regions in the brain may have great impact on the development of AD pathology.

Previous studies have demonstrated *C. pneumoniae *in both human and animal olfactory bulbs [8,33,34]. In both cell culture and animal studies, *C. pneumoniae *has been shown to infect nasal neuroepithelial cells [34]. In the animal studies, infection appeared to spread centrifugally from the vulnerable neuronal cells in the olfactory bulb into the brain [33,34,37]. Further, Chlamydia isolated from AD brains in a prior study was shown to have more commonalities with Chlamydia respiratory strains than with Chlamydia strains from atherosclerosis with the suggestion that the organism itself may have a tropism for specific cell types in the CNS [38]. Upon consideration of these data and our current data, a rationale for the selective vulnerability of specific brain regions to infection and resultant pathology emerges.

Notably, histopathological amyloid plaques and tangles are used to define the stage of AD, but the correlation with the pathology of the disease and the clinical manifestations of the disease are not always clear [2]. Some individuals who have massive pathology have little to no symptoms. On the other hand, some symptomatic individuals may show little pathology upon post-mortem histopathological examination. As such, there are many variations in the amount and type of damage evident in AD [1]. The variability of correlation between the symptomology and histopathology suggests other events and/or ingredients may be missing in the pathobiology of AD.

The response in the brain to infection may determine the extent of pathology and symptomatology that may arise. In this regard, *C. pneumoniae *infection characteristically promotes an inflammatory response whereby cytokines such as IL-1β and TNF-α are secreted and may initiate cellular damage [39]. These cytokine responses to infection parallel similar responses to amyloid accumulation [40]. Additionally, another cellular response to *C. pneumoniae *infection in culture is the production and processing of amyloid peptides. Labeling of infected cells in culture for Amyloid β 1-42 often reveals intracellular immunoreactivity at early post-infection times (unpublished observations CJH, DMA, CSL, BJB). Interestingly, our earlier study demonstrated intracellular and extracellular amyloid deposits in the brains of non-transgenic BALB/c mice following intranasal *C. pneumoniae *infection [34]. Our current study showed a similar relationship in which we demonstrated both amyloid and chlamydia immunoreactivity in the temporal cortex of the AD brain.

Further evaluation is required to specifically demonstrate how amyloid and *C. pneumoniae*, both intracellular and extracellular, are interrelated. In addition, future studies are required to further characterize the atypical extracellular Chlamydial immunolabeling profiles. Others have demonstrated evidence for an association of infection and amyloid in AD [41]. HSV1 viral DNA was shown to specifically associate with AD amyloid plaques [41]. Previous hypotheses have even suggested that amyloid in the AD brain may act as an antiviral agent [42] or an entrapping agent for infection [43]. Intriguingly, a recent study suggests that amyloid has anti-microbial properties, and may arise in response to brain infection in AD [44]. As 2 of 5 non-AD cases in our current study showed occasional chlamydia immunoreactivity and diffuse amyloid deposition, future analysis must also include mild cognitive impairment cases, as well as non-AD cases, as infection may be a prodromal event leading to eventual AD pathology.

## Conclusions

The exact consequences of infection that correlate best with the stage and subtype of Alzheimer's disease require ongoing investigation. This is true especially with regard to the hallmarks of the disease such as amyloid plaques and tau tangles. Alzheimer's disease is manifest with multifactorial aspects of pathology and with potentially multiple associated environmental factors, including infectious agents [8,10-12]. Further, our *Chlamydia pneumoniae *data suggest that the areas of the brain involved with olfaction are important to study as infection in these regions, as well as in the olfactory neuroepithelia and olfactory bulbs, may be a precursor to the pathology associated with AD. Identification and localization of infectious agents, such as *Chlamydia pneumoniae*, in specifically vulnerable areas and cell types in the brain is paramount. This determination may be a missing link in the current strategy of associating symptomatology to disease pathogenesis in sporadic late-onset Alzheimer's disease.

## Methods

### Human Post-mortem Samples

Post-mortem tissue samples from various brain regions (frontal and temporal cortices) of patients with and without AD were obtained through Dr. William Hill of the Medical College of Georgia (Augusta, GA), from the MCP-Hahnemann School of Medicine Department of Pathology, currently Drexel University College of Medicine (Philadelphia, PA), and the Alzheimer's Research Center of the Health Partners Research Foundation at Regions Hospital (St Paul, MN) under approved protocols at each procurement site. Five AD and 5 non-AD age-matched male and female archival brains were examined. All cases were confirmed as AD or non-AD by neuropathological examination at the source using standard diagnostic criteria (NINDS/CERAD) [45].

### Optimization of Immunohistochemistry

To detect chlamydia in the brain, IHC was performed on serial sections from formalin-fixed, paraffin-embedded Alzheimer brain tissue. Parallel IHC was performed using antibodies for AD amyloid plaque pathology, specifically anti-amyloid 1-42 antibodies. Amyloid plaques were best visualized when antigen retrieval was not performed whereas the optimal labeling for chlamydia antigens was achieved following antigen retrieval. Optimum immunoreactivity occurred at 37°C in a humidified chamber for both amyloid and chlamydial antigens. To visualize amyloid deposits, 3, 3' Diaminobenzidine (DAB) (golden brown color) was used. Red chromogens such as alkaline phosphatase (AP) red and AP new magenta were found to best visualize both intracellular and extracellular *C. pneumoniae*, as nothing in a typical brain section should appear red. For counterstaining, Mayer's hematoxylin was chosen as it only stains the nucleus leaving the cytoplasm of the cell clear allowing easier visualization of intracellular chlamydia EB and inclusion immunolabeling. Controls were used to rule out non-specific labeling. Our controls included: no primary or secondary antibodies, primary with no corresponding secondaries, no primary but HRP conjugated secondary and/or AP conjugated secondary alone and in combination, and anti-human IgG primary with appropriate HRP and/or AP secondaries alone and in combination, all of which were reacted with appropriate substrates DAB and/or AP red and/or AP magenta, alone and in combination. Controls for fluorescent work also included no primary, secondary, or stain to allow for evaluation of autofluorescence of the brain tissues.

### *Chlamydia pneumoniae *Immunohistochemistry

Archival, paraffin-embedded, human brain tissues were deparaffinized through xylenes and graded alcohols. Antigen retrieval was performed in The Retriever (Electron Microscopy Science, Fort Washington, PA) according to manufacturer's directions using 1 × Citra Antigen retrieval buffer (BioGenex, San Ramen, CA). The sections were rinsed with filtered water 3 × 5 min and treated with Alkaline Phosphatase/Horseradish Peroxidase Block (BioFX Laboratories, Owings Mills, MD) for 30 min at room temperature (RT). The tissues were rinsed with filtered water, PBS 3 × 5 min, and blocked in 1% Fetal Bovine Serum (FBS)/PBS for 5 min at RT. Sections were incubated in a humidified chamber with anti-chlamydia primary antibodies for 90 min at 37°C. Anti-chlamydial antibodies, genus and species specific, were purchased from commercial sources and used at dilutions shown in Table 1. Following the incubation, the sections were rinsed with PBS 3 × 5 min and blocked with 1% FBS/PBS block for 5 min at RT. The sections were incubated in a humidified chamber with appropriate anti-mouse or anti-rabbit Alkaline Phosphatase conjugated secondary antibody for 60 min at 37°C (BioFX Laboratories, Owings Mills, MD or Zymed- Invitrogen Corporation, Carlsbad, CA). After 3 × 5 min water rinses, the sections were reacted with BioFX AP-New Magenta IHC Substrate (BioFX Laboratories- SurModics, Owings Mills, MD) 40 min at RT or they were reacted with AP red (Zymed- Invitrogen Corporation, Carlsbad, CA) according to the manufacturer's directions. The sections were rinsed with water and counterstained with Mayer's hematoxylin (Electron Microscopy Science, Fort Washington, PA). The slides were aqueous mounted using Crystal Mount (Biomeda, Thermo Fisher Scientific, Pittsburgh, PA) and then permanently mounted using Permount (Thermo Fisher Scientific, Pittsburgh, PA). Sections were viewed on a Nikon Eclipse E800 or a Nikon Eclipse 90i microscope and captured using: the spot RT (Diagnostic Instruments, Starling Heights, MI) with the Image Pro Plus Phase 3 Imaging software (Media Cybernetics, Silver Spring, MD), the Nikon DS-Fi1 camera using the NIS-Elements Advanced Research version 3.0 software (Nikon) or the Nikon DS-Ri1 camera using the NIS-Elements Advanced Research version 3.0 software (Nikon).Depending on the available tissue, the IHC was performed on 2-3 serial sections for each antibody for each section of the brain that was available. Out of the battery of antibodies, 5 monoclonal and 1 polyclonal antibodies were more routinely used (Table 1). In order to be considered a positive sample, the sample must have been positive by IHC with at least 2 *C. pneumoniae *species-specific monoclonal antibodies in 2 of the 3 serial sections of each brain section. All chlamydia antibodies used on the tissue sections were also used on astrocyte, epithelial, monocyte, and/or neuronal cell lines that were uninfected or that had been infected with AR39 *Chlamydia pneumoniae *(ATCC, Manassas, VA) as antibody specificity controls.

### Amyloid Immunohistochemistry

Archival, paraffin-embedded, human brain tissues were deparaffinized through xylenes and graded alcohols. The sections were rinsed with filtered water and phosphate buffered saline (PBS) 3 × 5 min. Endogenous peroxidase was quenched using 3% H_2_O_2 _or Alkaline Phosphatase/Horseradish Peroxidase Block (BioFX Laboratories, Owings Mills, MD) for 30 min at RT. The tissues were rinsed with sterile filtered water, PBS 3 × 5 min, and blocked in 1% (FBS)/PBS for 5 min at RT. The primary antibody, mouse anti-human β-Amyloid (4G8, Signet, Covance, Cambridge, MA) or rabbit anti-Amyloid peptide β cleavage site 42 (Sigma, St Louis, MO), were incubated in a humidified chamber for 90 min at 37°C. Following the incubation, the sections were rinsed with PBS 3 × 5 min and blocked in 1% FBS/PBS block for 5 min at RT. The sections were incubated with anti-rabbit horseradish peroxidase conjugated (HRP) secondary antibody (Zymed, Invitrogen Corporation, Carlsbad, CA) in a humidified chamber for 60 min at 37°C. After a water rinse and PBS washes, 3 × 5 min, the sections were reacted with 3, 3′-Diaminobenzidine (DAB) (Sigma Aldrich, St Louis MI) for 10-20 min at RT. The sections were rinsed with water and counterstained with Mayer's hematoxylin (Electron Microscopy Science, Fort Washington, PA). Slides were dehydrated through graded alcohols and xylenes followed by cover slipping using Permount (Thermo Fisher Scientific, Pittsburgh, PA). Sections were viewed and captured as noted previously.

### *Chlamydia pneumoniae *antibody pre-absorption

Individual anti-chlamydia antibodies, mouse anti-*C. pneumoniae *(Table 1, #2, #3, BioDesign, Meridian Life Science, Inc., Saco, ME) or rabbit anti-chlamydia antibody (Table 1, #1, BioDesign, Meridian Life Science, Inc., Saco, ME), were mixed with >10 M excess of either Amyloid β 1-40 or Amyloid β 1-42 peptide (Sigma, St Louis, MO) in a batch method and allowed to incubate overnight at 4°C. The supernatant of each pre-absorbed antibody was then used for *Chlamydia pneumoniae *immunohistochemistry as described above with the exception that secondary antibodies were conjugated with HRP and reacted with DAB.

### Dual *Chlamydia pneumoniae *Immunohistochemistry and Thioflavin S staining

Slides immunolabeled for *C. pneumoniae *were stained using a modified Thioflavin S protocol http://www-medlib.med.utah.edu/WebPath/webpath.html[46]. In brief, following *Chlamydia pneumoniae *primary and subsequent secondary incubations and AP-New Magenta substrate development, slides were counterstained with Mayer's hematoxylin and coversliped with aqueous mount. Areas of chlamydia immunoreactivity were identified by light microscopy. The aqueous mount was then soaked off and the section were immersed in 1% Thioflavin S for 5 min, differentiated in 70% alcohol for 5 min, and coverslipped with water. The sections were kept wet by cover slipping with water as the intensity of the Thioflavin S was diminished with use of Crystal mount. Sections were viewed and captured while still wet using a Nikon Eclipse E800 microscope and Nikon DS-Ri1 camera. Light and fluorescent (under FITC filter ) images were captured and merged using the NIS-Elements Advanced Research version 3.0 software (Nikon).

### Quantitative analysis

For determination of amyloid and chlamydia labeling and the relationship between the two in the sections, we evaluated the relationship with the following quantification schemes.

For percentage of chlamydia pneumoniae immunopositiyity and percentage of type of immunopositivity, we first qualitatively determined the types of label to be counted: intracellular, extracellular or a combination of the two. In representative frontal and temporal sections, the total numbers of positives across a section were counted and then the numbers of each category tallied and the percentage of the total positive immunoreactivity was determined per total area across the section. Analysis at 4, 10 and 40× magnification was performed manually and utilizing the NIS-Elements Advanced Research version 3.0 software (Nikon). Using the software, the immunoreactivity was selected and the area or cells were counted. A similar quantification scheme was used in determining the relationship of the chlamydia immunoreactivity relative to the amyloid immunoreactivity across a representative temporal region. In this case, we again qualitatively determined the type of immunoreactivity: intracellular, extracellular, or a mixture of the two for amyloid and chlamydia. We then determined the counts and areas of single label and then the label of both in apposition to get an indication of the relative relationships between the two antigens

## Authors' contributions

CJH participated in design and coordination of experiments, prepared tissue samples, carried out immunohistochemistry experiments, analysis and drafted the manuscript.

LRH participated in immunohistochemistry experiments and helped to draft the manuscript.

RJH participated in immunohistochemistry experiments, quantitative analysis and data compilation.

CSL participated in design and pre-absorbed Chlamydia antibodies with amyloid peptides.

DMA participated in design and helped prepare tissue samples.

BJB conceived of the study, participated in design and analysis, prepared tissue samples, and helped draft the manuscript.

All authors read and approved of the final manuscript.

## References

[B1] Duyckaerts C Delatour B Potier MC Classification and basic pathology of Alzheimer disease Acta Neuropathol 2009 118 1 5 36 10.1007/s00401-009-0532-1 19381658

[B2] Savva GM Wharton SB Ince PG Forster G Matthews FE Brayne C Medical Research Council Cognitive Function and Ageing Study Age, neuropathology, and dementia N Engl J Med 2009 360 22 2302 2309 10.1056/NEJMoa0806142 19474427

[B3] Gouras GK Almeida CG Takahashi RH Intraneuronal Abeta accumulation and origin of plaques in Alzheimer's disease Neurobiol Aging 2005 26 9 1235 1244 10.1016/j.neurobiolaging.2005.05.022 16023263

[B4] D'Andrea MR Nagele RG Wang HY Lee DH Consistent immunohistochemical detection of intracellular beta-amyloid42 in pyramidal neurons of Alzheimer's disease entorhinal cortex Neurosci Lett 2002 333 322317315 163 16 10.1016/S0304-3940(02)00875-3 12429373

[B5] Takahashi RH Milner TA Li F Nam EE Edgar MA Yamaguchi H Beal MF Xu H Greengard P Gouras GK Intraneuronal Alzheimer abeta42 accumulates in multivesicular bodies and is associated with synaptic pathology Am J Pathol 2002 161 5 1869 1879 12414533 10.1016/s0002-9440(10)64463-x PMC1850783

[B6] Friedrich RP Tepper K Rönicke R Soom M Westermann M Reymann K Kaether C Fändrich M Mechanism of amyloid plaque formation suggests an intracellular basis of Aβ pathogenicity Proceedings of the National Academy of Sciences 2010 107 5 1942 1947 10.1073/pnas.0904532106 PMC2836607 20133839

[B7] Lambert MP Barlow AK Chromy BA Edwards C Freed R Liosatos M Morgan TE Rozovsky I Trommer B Viola KL Wals P Zhang C Finch CE Krafft GA Klein WL Diffusible, nonfibrillar ligands derived from Abeta1-42 are potent central nervous system neurotoxins Proc Natl Acad Sci USA 1998 95 11 6448 6453 10.1073/pnas.95.11.6448 9600986 PMC27787

[B8] Balin BJ Gerard HC Arking EJ Appelt DM Branigan PJ Abrams JT Whittum-Hudson JA Hudson AP Identification and localization of Chlamydia pneumoniae in the Alzheimer's brain Med Microbiol Immunol (Berl) 1998 187 1 23 42 10.1007/s004300050071 9749980

[B9] Miklossy J Alzheimer's disease--a spirochetosis? Neuroreport 1993 4 7 841 88 10.1097/00001756-199307000-00002 8369471

[B10] Miklossy J Khalili K Gern L Ericson RL Darekar P Bolle L Hurlimann J Paster BJ Borrelia burgdorferi persists in the brain in chronic lyme neuroborreliosis and may be associated with Alzheimer disease J Alzheimers Dis 2004 6 6 639 49 discussion 673-81 15665404 10.3233/jad-2004-6608

[B11] Itzhaki RF Lin WR Shang D Wilcock GK Faragher B Jamieson GA Herpes simplex virus type 1 in brain and risk of Alzheimer's disease [see comments] Lancet 1997 349 904797167222 241 244 10.1016/S0140-6736(96)10149-5 9014911

[B12] Gerard HC Dreses-Werringloer U Wildt KS Deka S Oszust C Balin BJ Frey WH Bordayo EZ Whittum-Hudson JA Hudson AP Chlamydophila (Chlamydia) pneumoniae in the Alzheimer's brain FEMS Immunol Med Microbiol 2006 48 3 355 366 10.1111/j.1574-695X.2006.00154.x 17052268

[B13] Michelow IC Olsen K Lozano J Rollins NK Duffy LB Ziegler T Kauppila J Leinonen M McCracken GH Jr Epidemiology and clinical characteristics of community-acquired pneumonia in hospitalized children Pediatrics 2004 113 4 701 77 10.1542/peds.113.4.701 15060215

[B14] Gieffers J Pohl D Treib J Dittmann R Stephan C Klotz K Hanefeld F Solbach W Haass A Maass M Presence of Chlamydia pneumoniae DNA in the cerebral spinal fluid is a common phenomenon in a variety of neurological diseases and not restricted to multiple sclerosis Ann Neurol 2001 49 5 585 589 10.1002/ana.1020 11357948

[B15] Campbell LA Kuo CC Chlamydia pneumoniae and atherosclerosis Semin Respir Infect 2003 18 1 48 54 10.1053/srin.2003.50006 12652454

[B16] Vikatmaa P Lajunen T Ikonen TS Pussinen PJ Lepantalo M Leinonen M Saikku P Chlamydial lipopolysaccharide (cLPS) is present in atherosclerotic and aneurysmal arterial wall--cLPS levels depend on disease manifestation Cardiovasc Pathol 2010 19 1 48 54 10.1016/j.carpath.2008.10.012 19150246

[B17] Bandaru VC Laxmi V Neeraja M Alladi S Meena AK Borgohain R Keerthi AS Kaul S Chlamydia pneumoniae antibodies in various subtypes of ischemic stroke in Indian patients J Neurol Sci 2008 272 1-2 115 122 10.1016/j.jns.2008.05.008 18571201

[B18] Du C Yao SY Ljunggren-Rose A Sriram S Chlamydia pneumoniae infection of the central nervous system worsens experimental allergic encephalitis J Exp Med 2002 196 12 1639 1644 10.1084/jem.20020393 12486106 PMC2196067

[B19] Sriram S Ljunggren-Rose A Yao SY Whetsell WO Jr Detection of chlamydial bodies and antigens in the central nervous system of patients with multiple sclerosis J Infect Dis 2005 192 7 1219 1228 10.1086/431518 16136465

[B20] Appelt DM Roupas MR Way DS Bell MG Albert EV Hammond CJ Balin BJ Inhibition of apoptosis in neuronal cells infected with Chlamydophila (Chlamydia) pneumoniae BMC Neurosci 2008 9 13 10.1186/1471-2202-9-13 18218130 PMC2266938

[B21] Boelen E Steinbusch HW van der Ven AJ Grauls G Bruggeman CA Stassen FR Chlamydia pneumoniae infection of brain cells: an in vitro study Neurobiol Aging 2007 28 4 524 532 10.1016/j.neurobiolaging.2006.02.014 16621171

[B22] MacIntyre A Abramov R Hammond CJ Hudson AP Arking EJ Little CS Appelt DM Balin BJ Chlamydia pneumoniae infection promotes the transmigration of monocytes through human brain endothelial cells J Neurosci Res 2003 71 5 740 750 10.1002/jnr.10519 12584732

[B23] MacIntyre A Hammond CJ Little CS Appelt DM Balin BJ Chlamydia pneumoniae infection alters the junctional complex proteins of human brain microvascular endothelial cells FEMS Microbiol Lett 2002 217 2 167 172 10.1111/j.1574-6968.2002.tb11470.x 12480099

[B24] Mahony JB Chlamydiae host cell interactions revealed using DNA microarrays Ann N Y Acad Sci 2002 975 192 201 10.1111/j.1749-6632.2002.tb05952.x 12538165

[B25] Zhong G Fan P Ji H Dong F Huang Y Identification of a chlamydial protease-like activity factor responsible for the degradation of host transcription factors J Exp Med 2001 193 8 935 942 10.1084/jem.193.8.935 11304554 PMC2193410

[B26] Pollack DV Croteau NL Stuart ES Uptake and intra-inclusion accumulation of exogenous immunoglobulin by Chlamydia-infected cells BMC Microbiol 2008 8 213 10.1186/1471-2180-8-213 19061499 PMC2621372

[B27] Fischer SF Hacker G Characterization of antiapoptotic activities of Chlamydia pneumoniae in infected cells Ann N Y Acad Sci 2003 1010 565 567 10.1196/annals.1299.105 15033792

[B28] Fischer SF Vier J Kirschnek S Klos A Hess S Ying S Hacker G Chlamydia inhibit host cell apoptosis by degradation of proapoptotic BH3-only proteins J Exp Med 2004 200 7 905 916 10.1084/jem.20040402 15452181 PMC2213288

[B29] van Zandbergen G Gieffers J Kothe H Rupp J Bollinger A Aga E Klinger M Brade H Dalhoff K Maass M Solbach W Laskay T Chlamydia pneumoniae multiply in neutrophil granulocytes and delay their spontaneous apoptosis J Immunol 2004 172 3 1768 1776 14734760 10.4049/jimmunol.172.3.1768

[B30] Stuart ES Troidle KM MacDonald AB Chlamydial glycolipid antigen: Extracellular accumulaton, biological activity, and antibody recognition 1994 28 2 85 90

[B31] Hybiske K Stephens RS Mechanisms of host cell exit by the intracellular bacterium Chlamydia Proc Natl Acad Sci USA 2007 104 27 11430 11435 10.1073/pnas.0703218104 17592133 PMC2040915

[B32] Fernandez-Obregon A Patton DL The role of Chlamydia pneumoniae in the etiology of acne rosacea: response to the use of oral azithromycin Cutis 2007 79 2 163 167 17388221

[B33] Little CS Bowe A Lin R Litsky J Fogel RM Balin BJ Fresa-Dillon KL Age alterations in extent and severity of experimental intranasal infection with Chlamydophila pneumoniae in BALB/c mice Infect Immun 2005 73 3 1723 1734 10.1128/IAI.73.3.1723-1734.2005 15731073 PMC1064908

[B34] Little CS Hammond CJ MacIntyre A Balin BJ Appelt DM Chlamydia pneumoniae induces Alzheimer-like amyloid plaques in brains of BALB/c mice Neurobiol Aging 2004 25 4 419 429 10.1016/S0197-4580(03)00127-1 15013562

[B35] Christen-Zaech S Kraftsik R Pillevuit O Kiraly M Martins R Khalili K Miklossy J Early olfactory involvement in Alzheimer's disease Can J Neurol Sci 2003 30 1 20 25 12619779 10.1017/s0317167100002389

[B36] Mann DM Tucker CM Yates PO Alzheimer's disease: an olfactory connection? Mech Ageing Dev 1988 42 1 1 15 10.1016/0047-6374(88)90058-9 2964546

[B37] Itzhaki RF Wozniak MA Appelt DM Balin BJ Infiltration of the brain by pathogens causes Alzheimer's disease Neurobiol Aging 2004 25 5 619 627 10.1016/j.neurobiolaging.2003.12.021 15172740

[B38] Dreses-Werringloer U Bhuiyan M Zhao Y Gerard HC Whittum-Hudson JA Hudson AP Initial characterization of Chlamydophila (Chlamydia) pneumoniae cultured from the late-onset Alzheimer brain Int J Med Microbiol 2009 299 3 187 201 10.1016/j.ijmm.2008.07.002 18829386 PMC2730674

[B39] Rasmussen SJ Eckmann L Quayle AJ Shen L Zhang YX Anderson DJ Fierer J Stephens RS Kagnoff MF Secretion of proinflammatory cytokines by epithelial cells in response to Chlamydia infection suggests a central role for epithelial cells in chlamydial pathogenesis J Clin Invest 1997 99 197148774 77 87 10.1172/JCI119136 9011579 PMC507770

[B40] Lue LF Brachova L Civin WH Rogers J Inflammation, A beta deposition, and neurofibrillary tangle formation as correlates of Alzheimer's disease neurodegeneration J Neuropathol Exp Neurol 1996 55 1097010972 1083 108 8858005

[B41] Wozniak MA Mee AP Itzhaki RF Herpes simplex virus type 1 DNA is located within Alzheimer's disease amyloid plaques J Pathol 2009 217 1 131 138 10.1002/path.2449 18973185

[B42] Kammerman EM Neumann DM Ball MJ Lukiw W Hill JM Senile plaques in Alzheimer's diseased brains: possible association of beta-amyloid with herpes simplex virus type 1 (HSV-1) L-particles Med Hypotheses 2006 66 2 294 299 10.1016/j.mehy.2005.07.033 16242250

[B43] Bishop GM Robinson SR Physiological roles of amyloid-beta and implications for its removal in Alzheimer's disease Drugs Aging 2004 21 10 621 630 10.2165/00002512-200421100-00001 15287821

[B44] Soscia SJ Kirby JE Washicosky KJ Tucker SM Ingelsson M Hyman B Burton MA Goldstein LE Duong S Tanzi RE Moir RD The Alzheimer's Disease-Associated Amyloid beta-Protein Is an Antimicrobial Peptide PLoS One 2010 5 3 e9505 10.1371/journal.pone.0009505 20209079 PMC2831066

[B45] Mirra SS Heyman A McKeel D Sumi SM Crain BJ Brownlee LM Vogel FS Hughes JP van Belle G Berg L The Consortium to Establish a Registry for Alzheimer's Disease (CERAD). Part II. Standardization of the neuropathologic assessment of Alzheimer's disease Neurology 1991 41 491187203 479 486 2011243 10.1212/wnl.41.4.479

[B46] WebPath: Internet Pathology Laboratory http://library.med.utah.edu/WebPath/HISTHTML/MANUALS/THIOFLAV.PDF

